# Caesarean delivery and its association with educational attainment, wealth index, and place of residence in Sub-Saharan Africa: a meta-analysis

**DOI:** 10.1038/s41598-022-09567-1

**Published:** 2022-04-01

**Authors:** Md.Akhtarul Islam, Nusrat Jahan Sathi, Md. Tanvir Hossain, Abdul Jabbar, Andre M. N. Renzaho, Sheikh Mohammed Shariful Islam

**Affiliations:** 1 Statistics Discipline, Science, Engineering and Technology School, Khulna University, Khulna, 9208 Bangladesh; 2 Sociology Discipline, Social Science School, Khulna University, Khulna, 9208 Bangladesh; 3 Department of Veterinary Medicine, Faculty of Veterinary Science, University of Veterinary and Animal Sciences, Lahore, Punjab Pakistan; 4 Translational Health Research Institute, School of Medicine, Western Sydney University, Penrith, NSW 2751 Australia; 5 Institute for Physical Activity and Nutrition, Deakin University, Melbourne, VIC 3125 Australia

**Keywords:** Health care, Risk factors

## Abstract

Caesarean delivery (C-section) has been increasing worldwide; however, many women from developing countries in Sub-Saharan Africa are deprived of these lifesaving services. This study aimed to explore the impact of certain socioeconomic factors, including respondent’s education, husband’s education, place of residence, and wealth index, on C-section delivery for women in Sub-Saharan Africa. We used pooled data from 36 demographic and health surveys (DHS) in Sub-Saharan Africa. Married women aged 15–49 years who have at least one child in the last five years were considered in this survey. After inclusion and excluding criteria, 234,660 participants were eligible for final analysis. Binary logistic regression was executed to determine the effects of selected socioeconomic factors. The countries were assembled into four sub-regions (Southern Africa, West Africa, East Africa, and Central Africa), and a meta-analysis was conducted. We performed random-effects model estimation for meta-analysis to assess the overall effects and consistency between covariates and utilization of C-section delivery as substantial heterogeneity was identified (I^2^ > 50%). Furthermore, the meta-regression was carried out to explain the additional amount of heterogeneity by country levels. We performed a sensitivity analysis to examine the effects of outliers in this study. Findings suggest that less than 15% of women in many Sub-Saharan African countries had C-section delivery. Maternal education (OR 4.12; CI 3.75, 4.51), wealth index (OR 2.05; CI 1.94, 2.17), paternal education (OR 1.71; CI 1.57, 1.86), and place of residence (OR 1.51; CI 1.44, 1.58) were significantly associated with the utilization of C-section delivery. These results were also consistent in sub-regional meta-analyses. The meta-regression suggests that the total percentage of births attended by skilled health staff (TPBASHS) has a significant inverse association with C-section utilization regarding educational attainment (respondent & husband), place of residence, and wealth index. The data structure was restricted to define the distinction between elective and emergency c-sections. It is essential to provide an appropriate lifesaving mechanism, such as C-section delivery opportunities, through proper facilities for rural, uneducated, impoverished Sub-Saharan African women to minimize both maternal and infant mortality.

## Introduction

As the millennium development goals (MDGs) ended in 2015, the sustainable development goals (SDGs) emerged with a purpose to lessen the maternal mortality ratio (MMR) to less than 70 per 100,000 live births worldwide by 2030^1^. With the enormous global attempt to restrain the threat of maternal and child death on account of the complicated pregnancy and delivery, maternal mortality is still unabated exclusively in the Sub-Saharan Africa (SSA) region^2^. The maternal mortality ratio is almost 14 times higher in developing countries than in developed countries worldwide, and the SSA region holds the maximum rates of maternal mortality^3^. Recently, it was projected that approximately 50% of total maternal mortality from pregnancy-related difficulties happened in Sub-Saharan Africa^4^. This high maternal mortality suggests the need to ensure evidence-based and high-quality maternal healthcare services, including advanced institutional delivery, skilled birth attendance, and standard approach utilization, such as caesarean section (C-section), to improve this crisis^5^.

To attain the goal three sustainable development goals, equality and equity inaccessibility to emergency obstetric care incorporating assisted vaginal delivery along with a safe C-section are tremendously crucial^6^. C-section is a surgical intervention designed to prevent or treat life-threatening maternal or foetal complications^7^. The potential to perform safe C-section delivery has been a significant improvement in obstetrics in the twentieth century^8^. Surgical deliveries are recommended exclusively in delivery complications where the health benefits of the intervention outweigh the risks^9^. However, there is no existence of standardized clinical algorithms for ascertaining the need for a C-section as the decision considers various factors and their changes over time. Consequently, if such a rate exists, which can be called ‘the optimal’ caesarean rate at the population level, it is unknown. A recent statement by the World Health Organization (WHO) on C-section calls for ‘every effort [to] be made to provide C-section to women in need, rather than striving to achieve a specific rate’^10^.

C-section has become a notable index of evaluating progress in emergency obstetric care and a medium to prevent complications during delivery and labour^11^. Accordingly, there is growing heed that C-section rates have typically been on the hike, regardless of race, medical condition, and gestational age, albeit unevenly. In developing countries, the C-section prevalence variation stretches from 2 to 39%^12,13^. According to the WHO, the developing world needed 3.2 million additional C-sections in 2008 while 6.2 million non-essential caesarean deliveries were performed elsewhere^14^. Global recognition over such upsurge has induced the WHO to recommend that C-section prevalence should not exceed 10–15%^15^. The previous evidence reveals that the prevalence of C-sections beyond 15% was not associated with reducing maternal and child mortality further^16^. An ecological study also deduced that in countries where the C-section rate was less than 15%, higher rates were analogous to the lower infant, neonatal, and maternal mortality rates^17^.

This is undoubtedly tough to narrate the role of C-section in poor-resource settings. Like the world at large, discrepancies exist in the C-section level in Sub-Saharan Africa (SSA) across varied socio-environmental and demographic factors, indicating inaccessibility to health care services^18^. Evidence shows that the low cost of C-sections is influencing people to prefer the C-section. A systematic review of the literature reveals that the C-section rate varies between 2 and 51% in SSA^19,20^. Another study stressing the aftermath of C-section in Africa noted that maternal mortality after caesarean delivery is 50 times higher than that of high-income countries and is driven by peripartum haemorrhage and anaesthesia complications^21^. The same study revealed that complications occurred in 17·4%, mainly were severe intraoperative and postoperative bleeding. Furthermore, maternal mortality was independently associated with a preoperative presentation of placenta praevia, placental abruption, ruptured uterus, antepartum haemorrhage and perioperative severe obstetric haemorrhage, or anaesthesia complications etc. Therefore, countries from SSA face a simultaneous double burden of both underuse and overuse of C-sections like the ‘too little, too late’ and ‘too much, too soon’ dichotomy perceived more extensively in delivery care^11^.

A previous study showed that the rate of C-section is consistently increasing due to diverse factors in many communities^22^. Women belonging to higher economic class with better education went through C-section more than women having a formal education and low economic level. Similarly, women who prefer private facilities over the government for delivery suffer more C-sections^23^. It is also well documented that women’s access to health care facilities with trained birth attendants (TBAs) together with sufficient drugs and supplies of other essentials significantly increased the likelihood of C-sections^24,25^. However, there is no denying that the additional burden to cover the associated expenses, i.e., medicines and emergency transports, on households could reduce the use of C-sections^25,26^. Therefore, maintaining the balanced use of C-sections is also very important for overuse, and less use of C-sections can lead to deleterious health outcomes^27^.

Several studies in the SSA region assessed the disparities among prevalence and influencing factors of C-section, its surgical site infections, and the effect of different government policies on C-section^28–31^. There are very few works that concentrate only on education, wealth, and place of residence that uses meta-analysis techniques using DHS data in the SSA region. This study identified the impact of these factors on the C-section delivery rate using meta-analysis techniques to estimate a more precise result in SSA.

## Methods

### Data source and extraction

**Figure 1:**
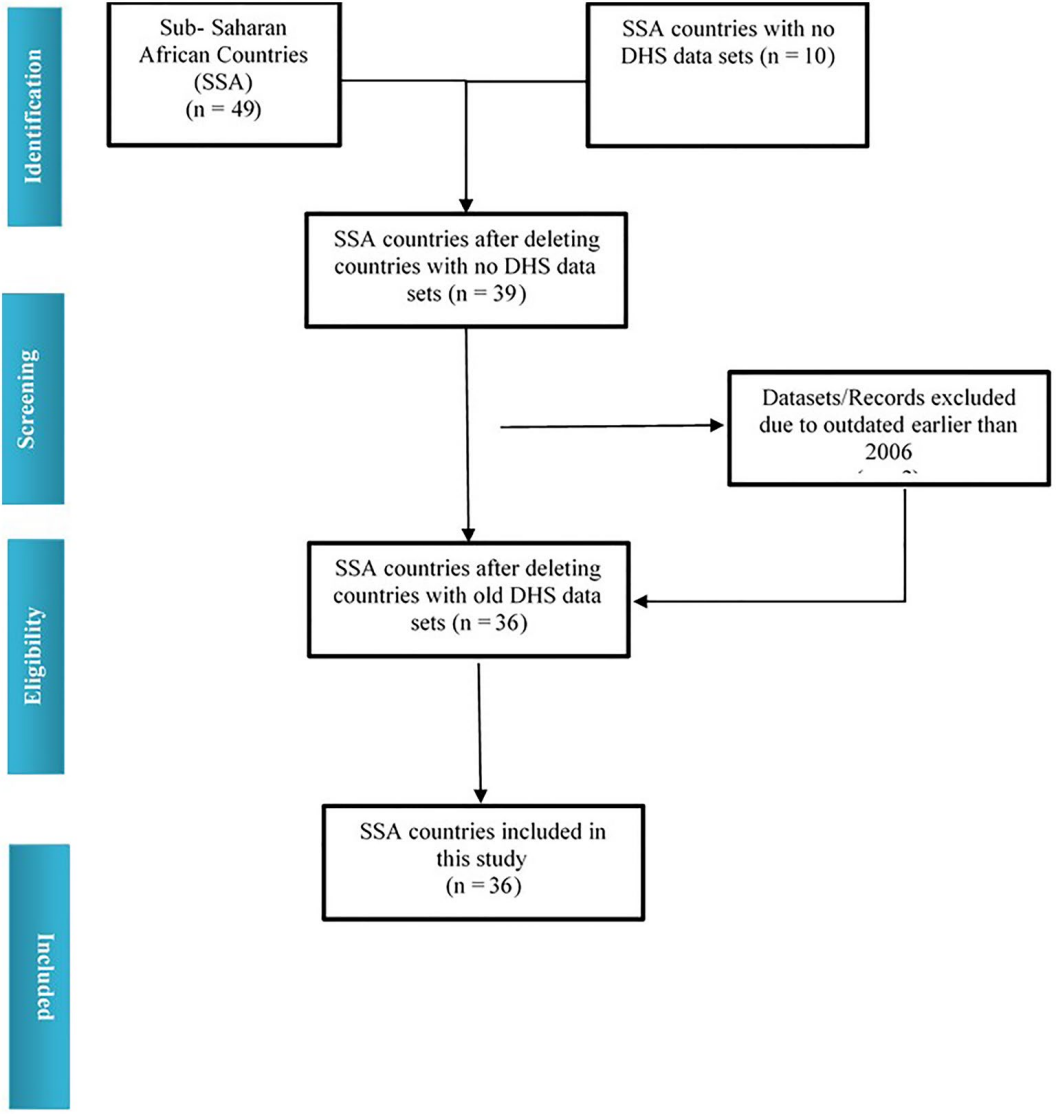
PRISMA (preferred reporting items for systematic reviews and meta-analysis) flow diagram illustrating the process of identifying and including DHS datasets for the random effect meta-analysis.

To collect Demographic and Health Survey (DHS) data, a cross-sectional study design is used for large nationally representative samples for every country. Information from the respondents was collected applying the same measures and similar questionnaires. In most of these surveys, a two-stage cluster sampling design with households in urban and rural strata has been used to choose the study respondents. Detailed information about the sampling and data collection methodology is available on the DHS websites^32^. The information utilized for this investigation was extracted from nationally representative secondary datasets of 36 developing countries (accessed in February 2021) from SSA^33^. The countries were Angola 2015–2016, Benin 2017–2018, Burkina Faso 2010, Burundi 2016–2017, Cameroon 2018, Chad 2014–2015, Comoros 2012, Congo Democratic Republic 2013–2014, Congo 2011–12, Cote d'Ivoire 2011–12, Eswatini 2006–2007, Ethiopia-2016, Gabon 2012, Gambia 2013, Ghana 2014, Guinea 2018, Kenya 2014, Lesotho 2014, Liberia 2013, Madagascar 2008–2009, Malawi 2015–2016, Mali 2018, Mozambique 2011, Namibia 2013, Niger 2012, Nigeria 2018, Rwanda 2014–2015, Sao Tome and Principe 2008–2009, Senegal 2010–2011, Sierra Leone 2019, South Africa 2016, Tanzania 2015–2016, Togo 2013–2014, Uganda 2016, Zambia 2018, Zimbabwe 2015. Married women of age 15–49 years having a minimum of 1 child during the last five years were considered in this survey. The DHS database contains information from 39 Sub-Saharan African countries (http://dhsprogram.com/data/available-datasets.cfm). We screened all the data set from SSA countries. We selected the data set from 2006 to 2021. We excluded 03 more countries before 2006. Finally, we have selected 36 SSA countries, where similar probability sampling was applied for data collection^33^ (Fig. 1). After excluding the missing cases, the participants from 36 SSA countries have been included in the study.

### Variables

The participants of this study were women who chose the C-section as delivery mode during their delivery; therefore, C-section delivery is the response variable. This response variable was categorized into two groups: those who had at least one C-section delivery were considered category ‘Yes’; those with no C-section were classified as ‘No’. For the logistic regression model, the independent variables include maternal education, paternal education, wealth index, place of residence. The categories of the place of residence (‘rural’, ‘urban’), respondent’s education (‘No education’, ‘Primary’, ‘Secondary’, ‘Higher’) and husband’s education (‘No education’, ‘Primary’, ‘Secondary’, ‘Higher’) remain same as it was in the BDHS data set. For the variable wealth index, were categorized it by adding poorer and poor to ‘poor’, middle as middle and rich, and richest to ‘rich’.

For the meta-analysis, we categorized parental education, including paternal and maternal, as ‘Educated’, merging primary, secondary, and higher education, and ‘Not educated’, including the not educated individuals. Wealth index was formulated into ‘Up to middle’ merging the poorest, poorer, and middle, whereas ‘Rich’ was constructed by combining richer and the richest. Place of residence the categories remained the same as it was found in the original dataset as rural and urban.

### Moderator

Birth by skilled health staff is recommended by healthcare centres worldwide for a healthy pregnancy period and childbirth. Renowned health organizations train the health staff to ensure safe and convenient delivery^34^. As a result, these attendants could appropriately assess the delivery mode and provide pregnancy-related awareness throughout gestation. More precisely, they have superior knowledge of dietary intake and subsequent pregnancy-related complications and treatments, enabling them to avoid various health risks. They directly affect childbirth mode, pregnancy care, and pregnancy-related consciousness^34^. Besides, the total percentage of births attended by skilled health staff (TPBASHS) of a country could be utilized to estimate the effect size and identify additional sources of heterogeneity between C-section delivery and socioeconomic variables; however, meta-regression is scarce on this dimension.

### Ethics declarations

The secondary source named Demographic Health Survey (DHS) was utilized to obtain the dataset. The only participant of the DHS program is human. We have got permission from https://dhsprogram.com/ to use the data through strict instructions and data confidentiality assurance. The DHS program has followed Helsinki Declaration (1964) ethical standards to assure ethical approval for each survey. The survey report from its website illustrates the entire survey information, including participants’ selection, sample size determination, ethical approval, sampling procedure, etc.

### Consent to participate

Each selected participant in the survey sampling was sent an informed consent before beginning the interview. It is because of ensuring participants’ authorization and confidentiality in the survey.

### Statistical analysis

We used statistical software SPSS V.23 (SPSS Inc. Chicago, USA) and R V.3.6.2 (Bell Laboratories, New Jersey, USA) to carry out the analysis. Binary logistic regression was executed using the key factors that impact C-section delivery using pooled data of 36 Sub–Saharan African countries^35,36^. We also applied meta-analysis techniques on the 36 DHS data from Sub –Saharan Africa^37^. Heterogeneity was assessed by enumerating values from I^2^ and *p* values among datasets^38,39^. We performed a random-effects model in the meta-analytical approach as significant heterogeneity was found by which we estimated DerSimonian and Laird's pooled effect^40,41^. The ‘leave-one-out’ sensitivity analysis was conducted to determine the effect of heterogeneity and outliers^42^. Forest plots were used to display 95% confidence interval (CI), 95% prediction interval, summary measure, and weight of each study for the most significant determinants^43^. The 95% prediction interval is used to estimate ranges that will define the expected effects for 95% of further studies. Subgroup analyses were applied to find out the regional difference of the influencing factors of C-section delivery across SSA countries.

Further, the authors deployed random-effects meta-regression with the total percentage of births attended by skilled health staff (TPBASHS) as a moderator in order to detect an extra amount of heterogeneity between C-section and socioeconomic variables, including education, place of residence, and wealth index. Test of moderator was performed by meta-regression to identify the significant influence of the moderator on effect size. The study is presented findings of meta-regression through scatterplot, executing in R Studio version 4.0.5. This study used ‘leave-one-country-out’ sensitivity analysis to assess the stability of the results and to examine whether one country had an excessive impact on the meta-analysis^44^. As a summary measure, we used Odds Ratio (OR), and all findings were weighted to handle bias due to under sampling and oversampling.

### Patient and public involvement

We have used nationally representative Demographic and Health Survey (DHS) secondary data sets. for Sub-Saharan African countries, which were collected following rigorous rules by the authorities. That is why any kind of patient and public involvement was not applicable for this study.

## Results

Table 1 shows the baseline characteristics for selected variables of 36 Sub-Saharan African countries. The proportion of delivery by caesarean section was lowest in Chad (1.3%); on the other hand, it was highest (24.5%) in South Africa among all countries.

**Table 1:** Baseline characteristics table for selected variables for different countries.

Country name	Respondent’s education n (%)		Wealth Index n (%)		Husband’s Education n (%)		Place of Residence n (%)		Delivery by caesarean (%)	
	Not educated	Educated	Up to middle	Rich	Not educated	Educated	Urban	Rural	No	Yes
Angola 2015–16	1491 (26.3)	4169 (73.7)	3508 (62.0)	2152 (38.0)	1115 (19.7)	4545 (80.3)	3550(62.7)	2110(37.3)	5411 (95.6)	249 (4.4)
Benin 2017–18	5262 (66.3)	2676 (33.7)	4937 (62.2)	3000 (37.8)	4358 (54.9)	3580 (45.1)	2989 (37.7)	4948 (62.3)	7478 (94.2)	460 (5.8)
Burkina Faso 2010	8558 (83.3)	1717 (16.7)	6409 (62.4)	3866 (37.6)	8325 (81.0)	1950 (19.0)	1881 (18.3)	8394 (81.7)	10,043 (97.7)	233 (2.3)
Burundi 2016–17	3676 (47.2)	4119 (52.8)	4960 (63.6)	2835 (36.4)	3012 (38.6)	4783 (61.4)	713 (9.1)	7083 (90.9)	7341 (94.2)	455 (5.8)
Cameroon, 2018	1544 (30.1)	3593 (69.9)	3368 (65.6)	1769 (34.4)	1251 (24.4)	3886 (75.6)	2238 (43.6)	2899 (56.4)	4950 (96.3)	188 (3.7)
Chad 2014–15	6820 (66.8)	3391 (8.8)	6362 (62.3)	3849 (37.7)	5986 (58.6)	4224 (41.4)	2005 (19.6)	8206 (80.4)	10,260 (98.7)	137 (1.3)
Comoros, 2012	848 (43.6)	1098 (56.4)	1248 (64.1)	698 (35.9)	725 (37.3)	1221 (62.7)	568 (29.2)	1378 (70.8)	1732 (89.0)	214 (11.0)
Congo Democratic Republic, 2013–14	1894 (18.7)	8245 (81.3)	6577 (64.9)	3562 (35.1)	786 (7.8)	9353 (92.2)	3121 (30.8)	7018 (69.2)	9561 (94.3)	578 (5.7)
Congo, 2011–12	317 (6.3)	4741 (93.7)	3148 (62.2)	1910 (37.8)	171 (3.4)	4887 (96.6)	3265 (64.5)	1794 (35.5)	4741 (93.7)	318 (6.3)
Cote d'Ivoire, 2011–12	2892 (65.9)	1497 (34.1)	2876 (65.5)	1513 (34.5)	2352 (53.6)	2037 (46.4)	1663 (37.9)	2726 (62.1)	4264 (97.1)	125 (2.9)
Eswatini, 2006–07	132 (10.3)	1146 (89.7)	773 (60.5)	505 (39.5)	176 (13.8)	1102 (86.2)	293 (22.9)	985 (77.1)	1167 (91.3)	111 (8.7)
Ethiopia-2016	4474 (63.3)	2592 (36.7)	4559 (64.5)	2507 (35.5)	3346 (47.4)	3720 (52.6)	872 (12.3)	6194 (87.7)	6903 (97.7)	163 (2.3)
Gabon, 2012	194 (7.5)	2389 (92.5)	1562 (60.5)	1021 (39.5)	204 (7.9)	2379 (92.1)	2212 (85.6)	371 (14.4)	2274 (88.0)	309 (12.0)
Gambia 2013	2947 (59.8)	1979 (40.2)	2999 (60.9)	1927 (39.1)	3042 (61.7)	1884 (38.3)	2411 (48.9)	2515 (51.1)	4808 (97.6)	118 (2.4)
Ghana, 2014	1035 (28.1)	2645 (71.9)	2220 (60.3)	1460 (39.7)	807 (21.9)	2873 (78.1)	1687 (45.8)	1993 (54.2)	3169 (86.1)	511 (13.9)
Guinea, 2018	3902 (77.9)	1105 (22.1)	3312 (66.2)	1694 (33.8)	3653 (73.0)	1353 (27.0)	1407 (28.1)	3600 (71.9)	4854 (97.0)	152 (3.0)
Kenya, 2014	654 (10.6)	5541 (89.4)	3592 (58.0)	2604 (42.0)	502 (8.1)	5693 (91.9)	2400 (38.7)	3796 (61.3)	5640 (91.0)	556 (9.0)
Lesotho, 2014	19 (0.8)	2231 (99.2)	1345 (59.8)	905 (40.2)	297 (13.2)	1953 (86.8)	648 (28.8)	1602 (71.2)	2017 (89.6)	233 (10.4)
Liberia, 2013	1710 (44.6)	2124 (55.4)	2534 (66.1)	1300 (33.9)	918 (23.9)	2916 (76.1)	1961 (51.1)	1873 (48.9)	3675 (95.9)	159 (4.1)
Madagascar, 2008–09	1845 (23.2)	6096 (76.8)	5155 (64.9)	2786 (35.1)	1733 (21.8)	6208 (78.2)	960 (12.1)	6981 (87.9)	7808 (98.3)	134 (1.7)
Malawi, 2015–16	1367 (12.4)	9643 (87.6)	6987 (63.5)	4023 (36.5)	1089 (9.9)	9921 (90.1)	1590 (14.4)	9420 (85.6)	10,316 (93.7)	693 (6.3)
Mali, 2018	4466 (73.0)	1650 (27.0)	3855 (63.0)	2261 (37.0)	4511 (73.7)	1606 (26.3)	1222 (20.0)	4895 (80.0)	5929 (96.9)	188 (3.1)
Mozambique, 2011	2545 (36.4)	4439 (63.6)	4521 (64.7)	2463 (35.3)	1801 (25.8)	5183 (74.2)	1897 (27.2)	5087 (72.8)	6694 (95.9)	290 (4.1)
Namibia, 2013	138 (7.5)	1708 (92.5)	1121 (60.8)	724 (39.2)	224 (12.1)	1622 (87.9)	994 (53.9)	851 (46.1)	1552 (84.1)	294 (15.9)
Niger, 2012	6636 (85.1)	1165 (14.9)	4721 (60.5)	3080 (39.5)	6387 (81.9)	1414 (18.1)	1062 (13.6)	6739 (86.4)	7674 (98.4)	127 (1.6)
Nigeria, 2018	9263 (45.8)	10,970 (54.2)	12,975 (64.1)	7258 (35.9)	7335 (36.2)	12,899 (63.8)	7922 (39.2)	12,311 (60.8)	19,590 (96.8)	643 (3.2)
Rwanda 2014–15	822 (15.2)	4588 (84.8)	3521 (65.1)	1889 (34.9)	919 (17.0)	4491 (83.0)	897 (16.6)	4514 (83.4)	4709 (87.0)	701 (13.0)
Sao Tome and Principe, 2008–09	9263 (45.8)	10,970 (54.2)	12,975 (64.1)	7258 (35.9)	7335 (36.2)	12,899 (63.8)	7922 (39.2)	12,311 (60.8)	19,590 (96.8)	643 (3.2)
Senegal, 2010–11	4804 (71.6)	1910 (28.4)	4277 (63.7)	2436 (36.3)	5033 (75.0)	1681 (25.0)	2586 (38.5)	4128 (61.5)	6258 (93.2)	456 (6.8)
Sierra Leone, 2019	3404 (58.7)	2399 (41.3)	3899 (67.2)	1904 (32.8)	3232 (55.7)	2571 (44.3)	1960 (33.8)	3843 (66.2)	5565 (95.9)	237 (4.1)
South Africa, 2016	28 (2.2)	1280 (97.8)	826 (63.1)	482 (36.9)	47 (3.6)	1262 (96.4)	962 (73.5)	347 (26.5)	988 (75.5)	321 (24.5)
Tanzania, 2015–16	1166 (20.5)	4516 (79.5)	3560 (62.7)	2122 (37.3)	752 (13.2)	4930 (86.8)	1569 (27.6)	4112 (72.4)	5284 (93.0)	398 (7.0)
Togo, 2013–14	1832 (40.4)	2708 (59.6)	2755 (60.7)	1785 (39.3)	1196 (26.3)	3344 (73.7)	1653 (36.4)	2886 (63.6)	4223 (93.0)	317 (7.0)
Uganda, 2016	860 (10.7)	7152 (89.3)	4956 (61.9)	3056 (38.1)	517 (6.5)	7495 (93.5)	1749 (21.8)	6263 (78.2)	7438 (92.8)	574 (7.2)
Zambia, 2018	512 (9.6)	4795 (90.4)	3352 (63.2)	1954 (36.8)	322 (6.1)	4985 (93.9)	1965 (37.0)	3342 (63.0)	5012 (94.5)	294 (5.5)
Zimbabwe, 2015	50 (1.2)	4113 (98.8)	2444 (58.7)	1718 (41.3)	54 (1.3)	4109 (98.7)	1345 (32.3)	2818 (67.7)	3899 (93.7)	264 (6.3)

Table 2 shows that the respondent from an urban area is 1.51 (OR 1.51; CI 1.44, 1.58) times more likely to use C-section delivery than the respondents forms rural areas. Respondents with high education were 4.12 times (OR 4.12; CI 3.75, 4.51) more likely to use C-section delivery than the respondents with no education. A respondent with a higher educated husband was 1.71 times (OR 1.71; CI 1.57, 1.86) more likely to use C-section delivery than the respondent with a not educated husband. A wealthy family respondent was 2.05 times (OR 2.05; CI 1.94, 2.17) more likely to use C-section delivery than a respondent from a low-income family.

**Table 2:** Results of the BLR model using pooled data of 36 Sub-Saharan African countries.

Variables	AOR	*p* value	95% CI for AOR		OR	*p* value	95% CI for OR	
			Lower	Upper			Lower	Upper
**Place of residence**								
Rural (ref)								
Urban	1.51	< 0.000	1.44	1.58	2.99	< 0.000	2.88	3.11
**Respondent’s education**								
No Education (ref)								
Primary	1.65	< 0.000	1.55	1.76	2.23	< 0.000	2.11	2.36
Secondary	2.02	< 0.000	1.89	2.16	3.82	< 0.000	3.62	4.04
Higher	4.12	< 0.000	3.75	4.51	11.07	< 0.000	10.32	11.87
**Husband’s education**								
No Education (ref)								
Primary	1.49	< 0.000	1.39	1.596	2.19	< 0.000	2.06	2.33
Secondary	1.42	< 0.000	1.33	1.528	3.16	< 0.000	2.98	3.34
Higher	1.71	< 0.000	1.57	1.862	6.88	< 0.000	6.44	7.35
**Wealth index**								
Poor (ref)								
Middle	1.32	< 0.000	1.24	1.41	1.66	< 0.000	1.56	1.77
Rich	2.05	< 0.000	1.94	2.17	4.04	< 0.000	3.85	4.23

*AOR* adjusted odds ratio, *CI* confidence interval, *OR* odds ratio; *ref* reference category.

Our study used the random-effects model as the study showed high between-study variations (heterogeneity). Table 3 shows the results of random effect model estimation results of 36 Sub-Saharan African countries, and Table 4 shows the summary effect of different explanatory variables. For respondent’s education, the overall OR was 2.55 (95% CI 2.14 to 3.05; p $\le$ 0.0001; 95% PI: 0.94 to 6.95), meaning the educated respondents were 155% more likely to use caesarian section delivery than the illiterate respondents. About 85.5% of the variation (I^2^ = 85.5%) was found for this variable. For husband’s education, the overall OR was 2.41 (95% CI 2.00 to 2.90; p $\le$ 0.0001; 95% PI: 0.84 to 6.92), meaning the respondents with educated husbands were 141% more likely to use C-section delivery than the respondents with illiterate husband (I^2^ = 89.3%). For the place of residence, the overall OR was 3.21 (95% CI 2.73 to 3.77; p $\le$ 0.0001; 95% PI: 1.22 to 8.46), which reveals the utilization of C-section delivery was 221% higher to the individuals who came from urban areas compared to those who were from rural settings (I^2^ 90.8%,). For wealth index, I^2^ was 87.8%, where the overall OR was 3.31 (95% CI 2.89 to 3.77; p $\le$ 0.0001; 95% PI: 1.52 to 7.20), which reveals that the utilization of C-section delivery was 231% higher for the individuals from rich families compared to the respondents from low-income families.

**Table 3:** Random-effects model estimation of odds ratio for different variables on 36 selected Sub- Saharan African countries.

Country(s)	Wealth index OR [95% CI]	Respondent’s education OR [95% CI]	Husband’s education OR [95% CI]	Place of residence OR [95% CI]
Angola 2015–2016	4.13 [3.13; 5.45]	3.86 [2.48; 6.01]	0.98 [0.71; 1.34]	4.74 [3.21; 7.01]
Benin 2017–2018	3.72 [3.04; 4.54]	2.71 [2.24; 3.28]	3.06 [2.49; 3.75]	2.49 [2.05; 3.01]
Burkina Faso 2010	4.43 [3.32; 5.92]	3.53 [2.70; 4.62]	3.21 [2.46; 4.18]	6.31 [4.84; 8.22]
Burundi 2016–2017	2.94 [2.42; 3.57]	1.72 [1.41; 2.10]	1.90 [1.53; 2.36]	5.02 [4.04; 6.25]
Cameroon 2018	5.31 [3.84; 7.35]	10.13 [4.98; 20.62]	3.84 [2.26; 6.53]	3.96 [2.84; 5.53]
Chad 2014–2015	2.27 [1.64; 3.13]	2.46 [1.78; 3.39]	3.15 [2.24; 4.45]	5.44 [3.94; 7.52]
Comoros 2012	2.14[1.59; 2.87]	2.61 [1.85; 3.69]	2.82 [1.93; 4.12]	1.58 [1.17; 2.12]
Congo Democratic Republic 2013–2014	2.21 [1.87; 2.62]	1.05 [0.84; 1.31]	0.63 [0.48; 0.83]	1.87 [1.58; 2.22]
Congo 2011–2012	2.50 [1.98; 3.15]	2.13 [1.12; 4.05]	3.86 [1.22; 12.15]	2.90 [2.15; 3.92]
Cote d'Ivoire 2011–2012	6.08 [4.03; 9.17]	2.90 [2.02; 4.16]	2.37 [1.63; 3.44]	4.99 [3.32; 7.49]
Eswatini 2006–2007	1.30 [0.89; 1.90]	2.83 [1.13; 7.06]	1.79 [0.92; 3.49]	1.27 [0.84; 1.92]
Ethiopia-2016	5.83 [4.07; 8.36]	6.59 [4.52; 9.61]	4.66 [3.08; 7.07]	14.48 [10.42; 20.13]
Gabon 2012	2.86 [2.24; 3.66]	0.91 [0.59; 1.41]	2.28 [1.26; 4.14]	1.78 [1.19; 2.67]
Gambia 2013	2.35 [1.62; 3.41]	1.48 [1.03; 2.13]	1.61 [1.12; 2.31]	2.33 [1.57; 3.45]
Ghana 2014	3.79 [3.11; 4.62]	3.09 [2.35; 4.05]	3.71 [2.67; 5.16]	2.69 [2.21; 3.28]
Guinea 2018	4.59 [3.24; 6.51]	2.98 [2.15; 4.13]	2.19 [1.58; 3.03]	4.77 [3.41; 6.68]
Kenya 2014	3.37 [2.79; 4.06]	4.61 [2.74; 7.75]	4.72 [2.58; 8.64]	2.53 [2.12; 3.02]
Lesotho 2014	2.14 [1.63; 2.82]	2.09 [0.28; 15.72]	3.03 [1.67; 5.49]	1.60 [1.21; 2.12]
Liberia 2013	2.63 [1.86; 3.71]	1.84 [1.30; 2.59]	1.54 [1.01; 2.34]	2.40 [1.73; 3.34]
Madagascar 2008–09	8.71 [5.58; 13.58]	6.57 [2.89; 14.93]	7.27 [2.97; 17.80]	6.83 [4.83; 9.65]
Malawi 2015–16	2.64 [2.26; 3.08]	2.22 [1.62; 3.04]	1.71 [1.24; 2.34]	2.49 [2.09; 2.96]
Mali 2018	2.48 [1.84; 3.33]	1.75 [1.30; 2.36]	2.56 [1.91; 3.43]	2.77 [2.05; 3.73]
Mozambique 2011	4.48 [3.47; 5.78]	3.23 [2.34; 4.46]	1.99 [1.44; 2.75]	4.07 [3.21; 5.18]
Namibia 2013	5.04 [3.83; 6.64]	2.55 [1.32; 4.92]	2.35 [1.43; 3.86]	3.68 [2.74; 4.94]
Niger 2012	1.81 [0.81; 4.04]	3.38 [1.88; 6.05]	0.56 [0.13; 2.32]	6.78 [3.60; 12.78]
Nigeria 2018	7.31 [6.03; 8.87]	8.17 [6.29; 10.61]	9.74 [6.98; 13.59]	4.36 [3.66; 5.20]
Rwanda 2014–15	2.26 [1.93; 2.66]	1.78 [1.37; 2.31]	1.46 [1.15; 1.84]	2.31 [1.93; 2.78]
Sao Tome and Principe 2008–09	1.24 [0.72; 2.13]	1.26 [0.49; 3.22]	3.91 [1.45; 10.56]	2.01 [1.24; 3.25]
Senegal 2010–11	4.52 [3.68; 5.55]	2.97 [2.45; 3.59]	3.32 [2.74; 4.03]	4.24 [3.44; 5.22]
Sierra Leone 2019	2.53 [1.95; 3.28]	1.38 [1.06; 1.79]	1.37 [1.06; 1.78]	2.57 [1.98; 3.34]
South Africa 2016	3.11 [2.40; 4.03]	2.75 [0.83; 9.18]	2.21 [0.93; 5.26]	1.49 [1.10; 2.02]
Tanzania 2015–2016	3.81 [3.07; 4.74]	2.92 [2.04; 4.17]	2.88 [1.84; 4.50]	3.34 [2.71; 4.10]
Togo 2013–2014	4.31 [3.35; 5.55]	2.53 [1.92; 3.32]	3.79 [2.57; 5.59]	3.54 [2.79; 4.50]
Uganda 2016	3.32 [2.78; 3.96]	1.98 [1.39; 2.83]	1.88 [1.21; 2.94]	2.73 [2.29; 3.26]
Zambia 2018	3.64 [2.84; 4.67]	1.34 [0.86; 2.08]	1.21 [0.71; 2.07]	3.23 [2.53; 4.13]
Zimbabwe 2015	3.48 [2.66; 4.55]	1.63 [0.39; 6.76]	3.62 [0.50; 26.31]	3.59 [2.78; 4.64]
${\mathrm{I}}^{2}$	87.8% [84.1; 90.6]	88.5% [85.1; 91.1]	89.3% [86.2; 91.7]	90.8% [88.3; 92.8]
${\uptau }^{2}$	0.1140	0.2268	0.2780	0.1644
95% PI	[1.52; 7.20]	[0.94; 6.95]	[0.84; 6.92]	[1.22; 8.46]

*OR* odds ratio, *CI* confidence interval, *PI* prediction interval.

**Table 4:** Random-effects model estimation (summary effect) for different variables on 36 selected Sub- Saharan African countries.

Variables	Random effects model			
	OR	*p* value	95% CI	
			Lower bound	Upper bound
Respondent’s education	2.55	0.0001	2.14	3.05
Husband’s education	2.41	0.0001	2.00	2.90
Place of residence	3.21	0.0001	2.73	3.77
Wealth index	3.31	0.0001	2.89	3.77

*OR* odds ratio, *CI* confidence interval.

**Figure 2:**
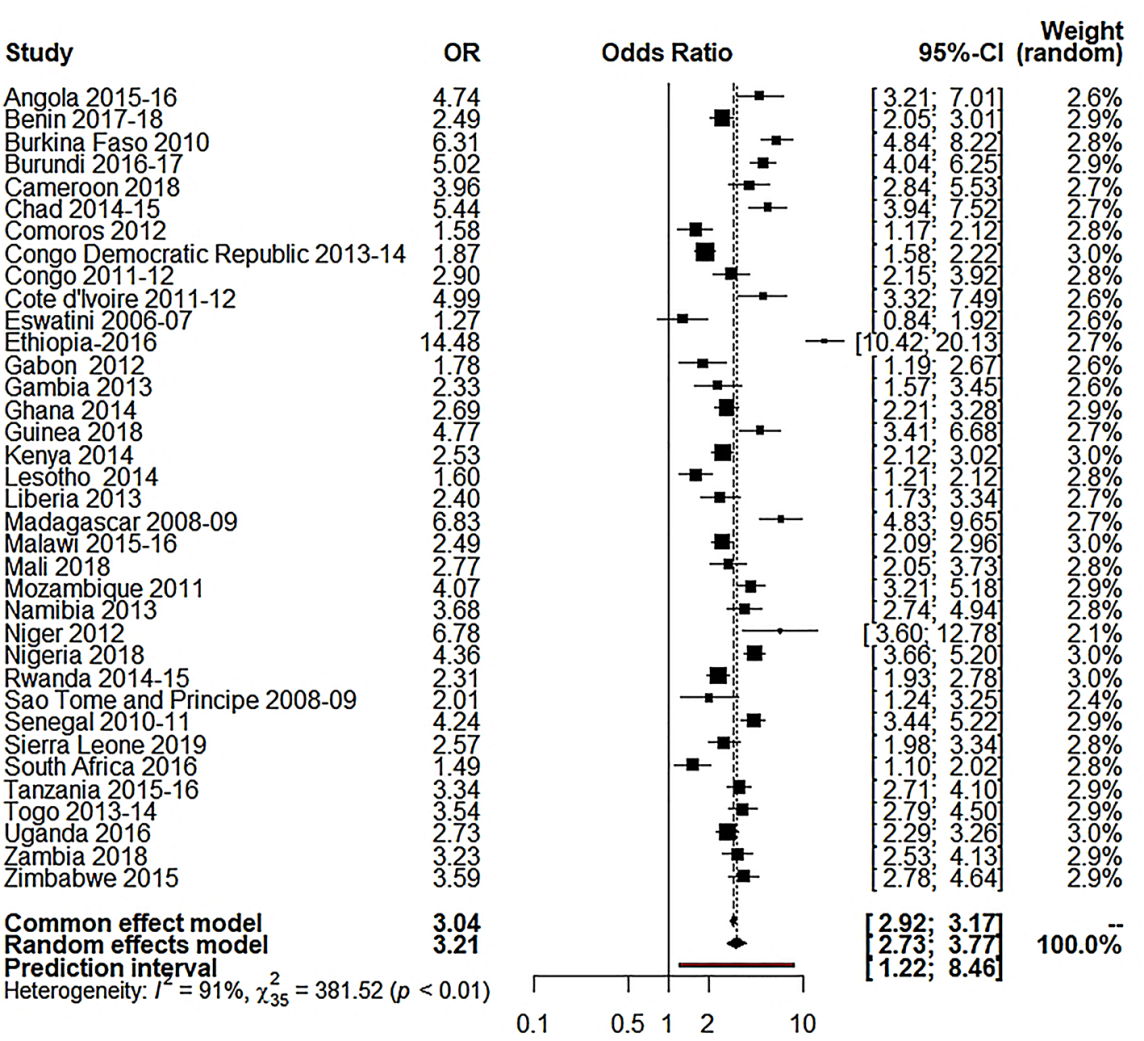
Forest plot for the place of residence.

**Figure 3:**
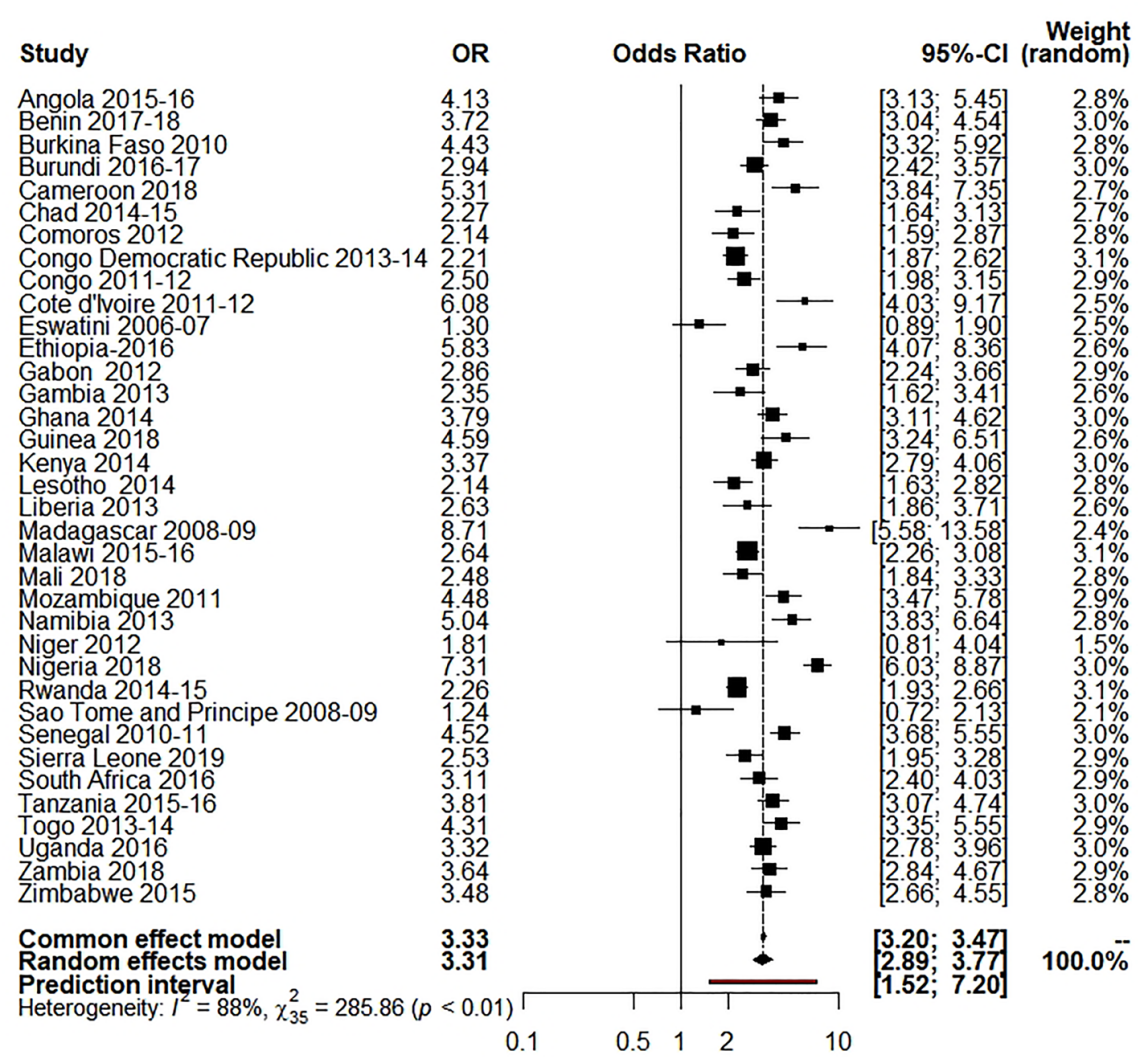
Forest plot for wealth index.

Table 5 and Fig. 2 show that place of residence significantly affected the C-section delivery concerning the region in SSA countries. For instance, women who lived in urban areas were more likely to utilize C-section delivery in the East Africa region (OR 3.56, p < 0.01, I^2^ = 94%) than the other regions of SSA countries. Figure 3 and Table 5 show that the respondents from a rich family background were more likely to take C-section delivery. The West Africa region has the highest tendency of C-section (OR 3.66, p < 0.01, I^2^ = 87%) than the other SSA regions.

**Table 5:** Sub-group analysis for different factors.

Variables	Central Africa		West Africa		East Africa		Southern Africa	
	OR (95%CI)	*p* value ${I}^{2}$	OR (95%CI)	*p* value ${I}^{2}$	OR (95%CI	*p* value ${I}^{2}$	OR (95%CI	*p* value ${I}^{2}$
Place of residence	3.14 [2.24; 4.43]	< 0.01 89%	3.45 [2.85; 4.18]	< 0.01 84%	3.56 [2.76; 4.60]	< 0.01 94%	1.84 [1.15; 2.97]	< 0.01 89%
Wealth index	2.92 [2.25; 3.79]	< 0.01 86%	3.66 [2.95; 4.55]	< 0.01 87%	3.46 [2.93; 4.09]	< 0.01 85%	2.60 [1.55; 4.35]	< 0.01 92%
Respondent’s education	2.26 [1.37; 3.72]	< 0.01 82%	2.64 [2.01; 3.47]	< 0.01 91%	2.65 [2.03; 3.47]	< 0.01 84%	2.63 [1.64; 4.22]	< 0.01 0.00%
Husband’s education	2.08 [1.20; 3.62]	< 0.01 92%	2.70 [2.01; 3.63]	< 0.01 91%	2.37 [1.85; 3.04]	< 0.01 78%	2.35 [1.73; 3.21]	< 0.01 0.00%

*Q* heterogenic statistic, *I*^*2*^ Between study variation, *OR* odds ratio, *CI* confidence interval.

**Figure 4:**
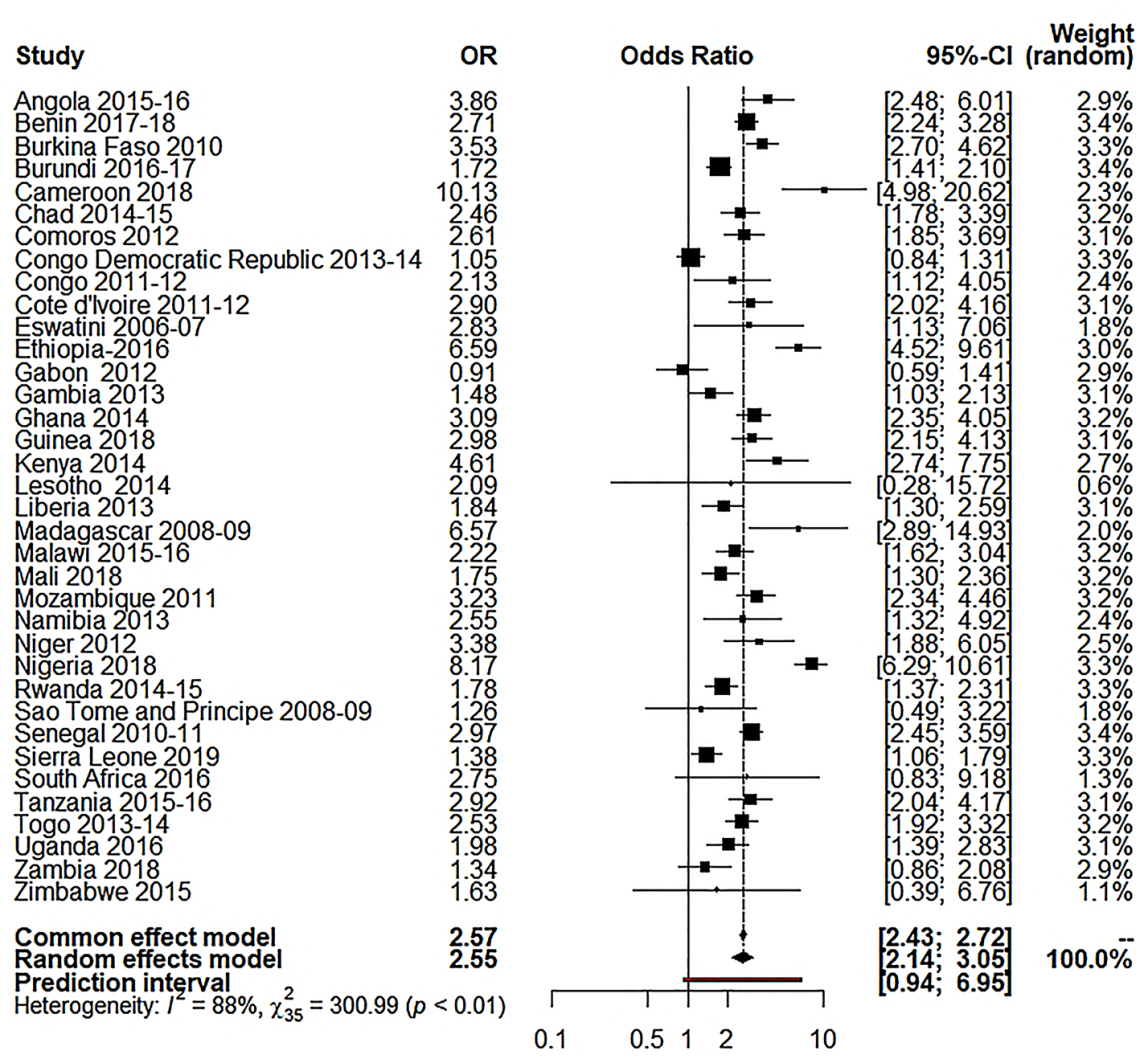
Forest plot for respondent’s education.

**Figure 5:**
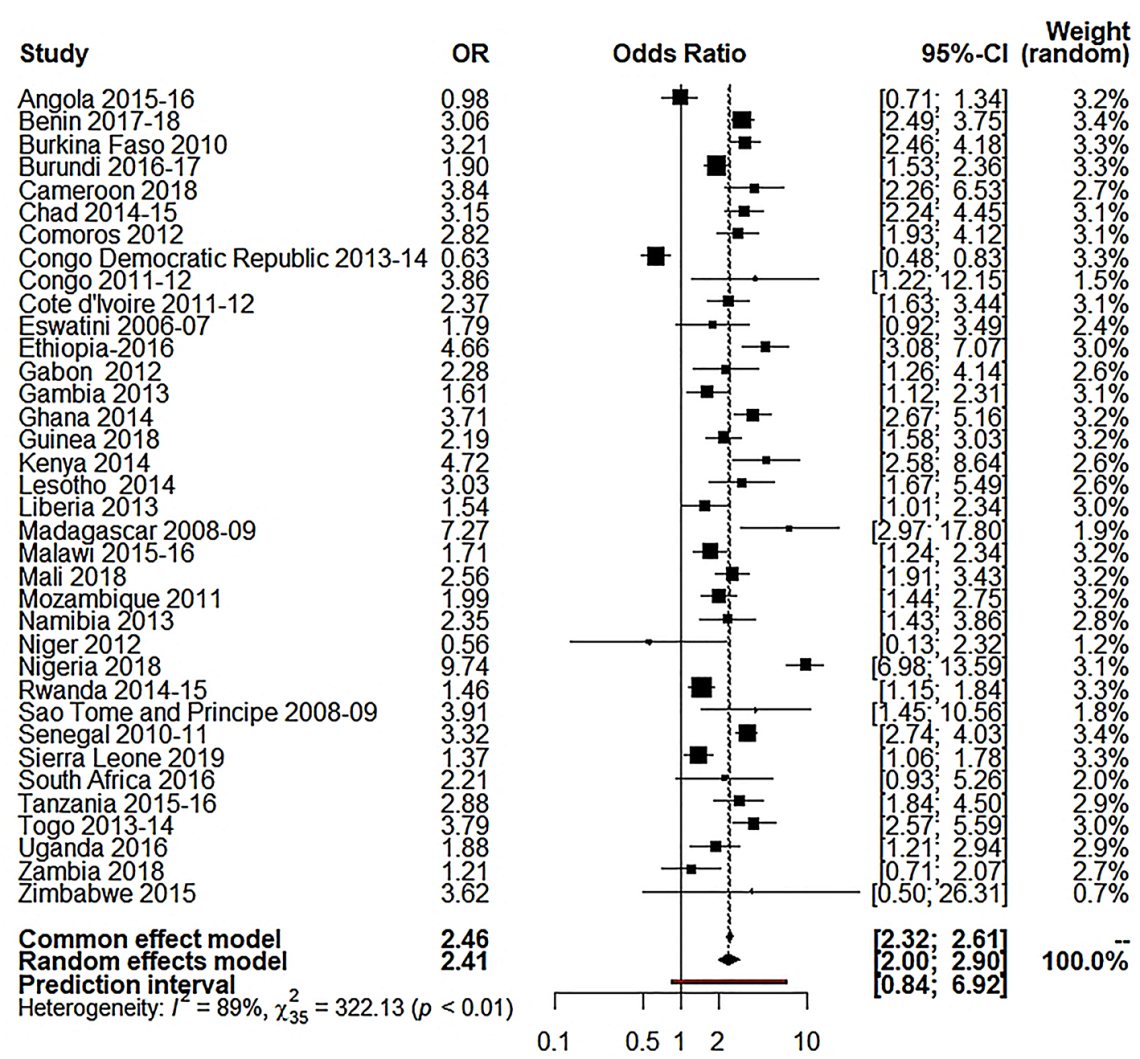
Forest plot for husband’s education.

Figure 4 indicates that the respondent’s education significantly influenced the C-section delivery in SSA countries. For instance, educated women were more likely to utilize C-section delivery in the East Africa region (OR 2.65, p < 0.01, I^2^ = 84%) than the other regions of SSA countries. In Fig. 5, it is clear that the husband’s education significantly influenced the utilization of C-sections across the SSA countries. Results indicated that the respondents with educated husbands were more prone to use C-section delivery in West Africa (OR 2.71, < 0.01, I^2^ = 91%).

Tests of moderator for educational attainment, residence, and wealth index are exhibited in Table 6. The findings reveal that these tests are statistically significant for all socioeconomic variables. Consequently, TPBASHS has a significant influence on the countries’ effect size and could significantly explain the extra amount of heterogeneity for all four variables.

**Table 6:** Output of test of moderator for different variables on 36 selected Sub- Saharan African countries.

Variables	Respondent education	Husband education	Place of residence	Wealth index
Moderator	TPBASHS	TPBASHS	TPBASHS	TPBASHS
Estimate	18.55	4.70	37.09	14.55
*P*-value	< 0.0001	0.0302	< 0.0001	0.0001

*TPBASHS* total percentage of births attended by skilled health staff.

**Figure 6:**
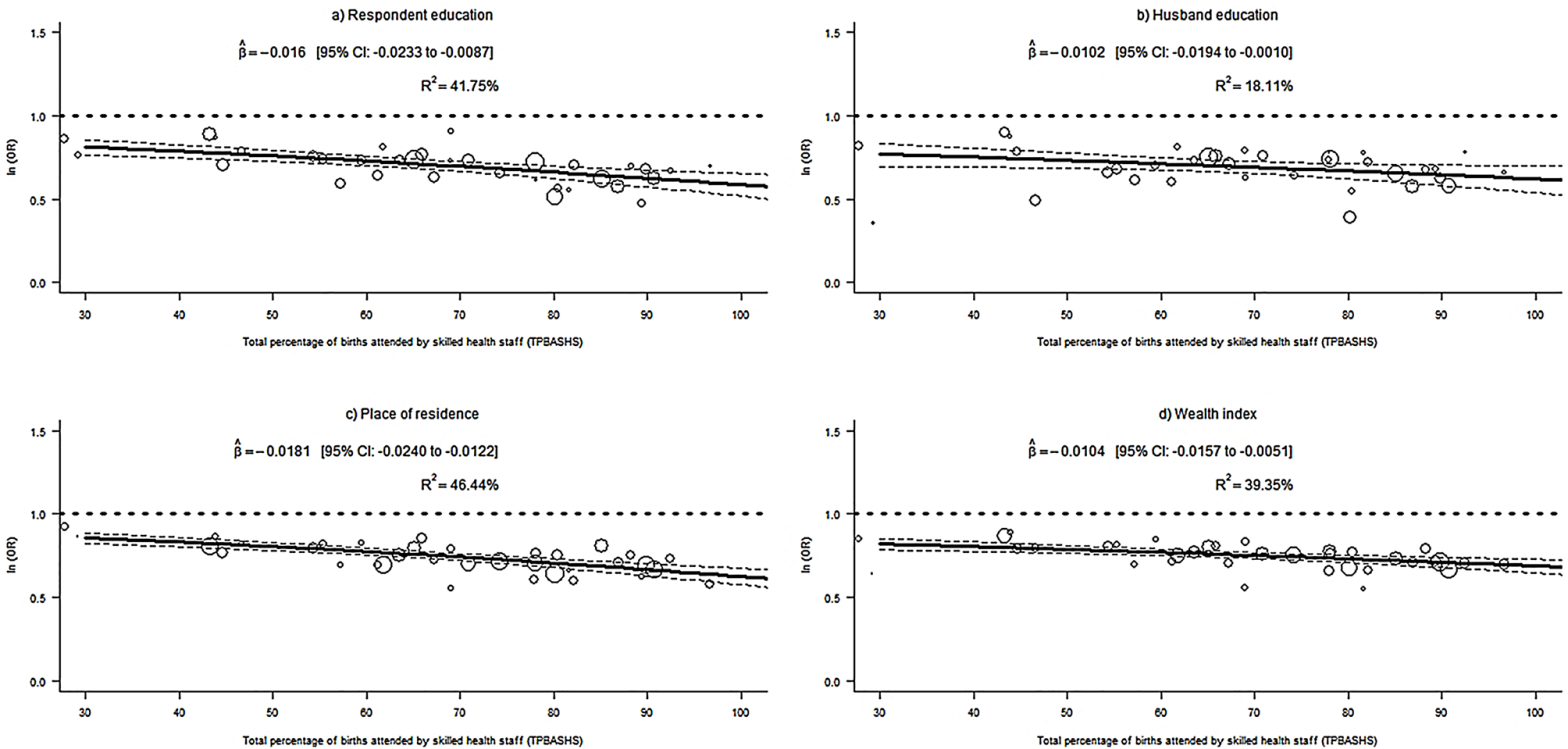
Meta-regressions of C-section delivery and socio-economic factors, (**a**) respondent education: TPBASHS (**b**) husband education: TPBASHS (**c**) place of residence: TPBASHS (**d**) wealth index: TPBASHS.

Figure 6 shows the odds of C-section utilization to determine the influence of TPBASHS in all 36 countries from the Sub-Saharan African sub-continent. A significant negative association is observed between TPBASHS and C-section delivery for all four socioeconomic variables. For respondent educational attainment and accommodation, the estimated value of TPBASHS were -0.0160 (95% CI − 0.0233 to − 0.0087; *R*^*2*^ = 41.75%) and − 0.0181 (95% CI − 0.0240 to − 0.0122; *R*^*2*^ = 46.44%). The findings also include that 41.75% (respondent education) and 46.44% (place of residence) of variation in true effect sizes could be explained by TPBASHS.

## Discussion

Over past decades, an uncertain increase in C-section rate is occurring. Education, wealth, and residence were strongly associated with the utilization of C-section in the SSA regions. The current study was more informative in providing the rate and association of C-section delivery with education, wealth, and place of residence as compared to that of previous findings. In the current study, the highest caesarean rate was found in Latin America and the Caribbean regions (40.5%), followed by Northern America (32.3%), Oceania (31.1%), Europe (25%), Asia (19.2%), and Africa (7.3%). This trend is increasing worldwide, especially in Egypt, Turkey, Dominican Republic, Georgia, and China, except for two countries (Guinea and Nigeria). The C-section rate decreased in Zimbabwe. These different rates of C-section in different countries showed that it was varying widely among the SSA countries, while WHO recommends that it should be 5–15%^45^. Dikete et al. reported that C-section rate in sub-Saharan Africa was 14 to 24%, which showed variation from the current study^46^. Bonsaffoh et al. reported a higher level of C-section rate i.e., 46%, as compared to current findings^47^. Betran et al. reported a study similar to the present finding in which 18.6% of all births occurred by C-section. In that previous finding, the highest caesarean rate was in Latin America, Caribbean regions, and Northern America (42.9%) followed by northern Africa (27.8%), Africa (7.3%), Saharan Africa (3.5%). Sanni et al. reported a study dissimilar to the present study in which the public health care centres rate of C-section was 3% in Burana and 15.6% in Ghana. While the private health care centers were having a C-section rate of 0% and 64% in Sao tome and Rwanda^48^.

A metanalysis on 36 different countries of sub-Saharan showed that 41.75%, 18.11%, 46.44%, and 36.35% of the C-section deliveries occur due to respondent education, husband education, place of residence, and wealth index, respectively. The BLR model and meta-analysis revealed that parents’ education is a crucial factor that is the reason behind the increased rate of C-sections. Educated respondents are more likely to use C-section delivery compared to respondents with no education. Regarding the association of C-section rate with education, the current study showed similarity with many of the previous studies except a few ones. Majid et al. (2019) presented a study in Iran that showed that the probability of utilizing the C-section is higher among educated women than women having lower education^49^. Arendt et al. (2017), Feng et al. (2020), Nababan et al. (2018), and Divyamol et al. (2016) also indicated that an increase in maternal education level leads to C-section delivery as educated women make their own decision regarding delivery, and they, in general, prefer C-section delivery^50–53^. However, previous findings of Epistein et al., Lee et al., and Mumtaz et al. reported that the educated women prefer C-section as the level of pain is lower, and they believe it is safer and interferes less with the workload, leisure time, and socially more prestigious than the normal vaginal delivery^49,54,55^. Another study by Long et al. (2015) showed that the overall OR for paternal education also showed that husbands with education are more likely to use C-section delivery than husbands with no education. The result delivered a similar statement from the BLR model and was consistent with previous studies^56^. A study reported by Bandori et al. showed exception. It showed that high education is not always positively associated with the likelihood of having the C-section. Therefore, in South Korea, the negative association between education and C-section rate has also been seen in the literature. The reason for this exception is that the education is also providing information on health-promoting behaviour, more educated women have more knowledge about the risk of unnecessary C-sections^57–59^.

The second determent highlighted by this study was the place of residence that plays an essential role in the increased C-section rate. The utilization of C-section delivery is higher to individuals from urban areas, whereas the rate is comparatively lower to individuals from rural settings. Similar to the current study, a previous study reported by Bahadori et al. (2013) showed that the women from rural areas had less chance to utilize the C-section delivery than women from urban areas^57^. Another study showed similarity with the current study by describing the prevalence of C-sections rates ranging from 4.6% to 12.2% for urban areas, whereas the prevalence of C-section rate ranging from 1.6% to 3.9% for rural areas^60^. The findings of this study are also in line with the previous studies, which suggest that the prevalence of C-sections in urban areas is significantly higher than those in rural areas. Maybe women who live in rural areas have fewer delivery mode options, awareness, and limited financial resources^58,61,62^. Based on 80 demographic and health surveys conducted in 26 countries in Southern Asia and SSA, a study reported by Cavallaro et al. (2013) revealed that women from urban areas were more likely to utilize the C-section delivery than rural women^59^. Availability, accessibility, and affordability of C-sections in urban areas women are more than the rural women, more private C-section facilities and higher women employment rate in urban areas could be the reasons behind the high caesarean rate in urban areas compared to rural areas^63,64^.

Wealth status is another determinant of increased C-section. Wealthy families utilize C-sections more than low-income families, which is in line with current findings. Previous studies reported that the C-section tendency is more among women with a wealthy family background than women with low-income family background^55,65–67^, and the other study reveals that 12.3% of the rich women had a C-sections delivery compared to 1.7% of the poor women^60^. Different studies suggested that poor women in the developing world cannot afford this lifesaving procedure and give birth at home, which is another reason for wealthy family women's high caesarean delivery in SSA^48,68,69^. A data analysis based on over 20,000 births shows that women of higher socioeconomic backgrounds who had better access to antenatal services are the most likely to undergo a C-section^70^. However, the opposite trend has also been observed in developed countries, where higher education and economic status were protective against C-sections as awareness and knowledge of childbirth are expected to be high among this group of women^71^.

### Strength and limitation

This study used a large sample size involving multiple nationally representative datasets from 36 countries in SSA to investigate the prevalence of C-sections in the sub-Sahara Africa region. We combined two methods: binary logistic regression and meta-analysis of 36 DHS data of Sub-Saharan African countries. The integrated findings enlarged the validity of the outcome of the research. We unfolded a new research approach by introducing this mixed-method design. Because of its extensive and acute quality, better knowledge and insights could be generated. Also, we applied subgroup analysis to find out the regional effect.

Nonetheless, the data lacks information relating to clinical indications for C-sections, as the data did not distinguish between elective and emergency C-sections. Also, the use of this information for decision-making and comparison should consider the cross-sectional nature of the data, which is inadequate to sufficiently establish causality. Another limitation is that for estimating OR from random-effects meta-analysis, we had to create 2 × 2 cross-tabulation for which each variable was categorized into two categories only. Moreover, a vast number of factors that could influence C-sections could not be included in our study because the DHS has very limited information on both supply- and demand-side variables regarding C-section.

## Conclusion

The findings from the present study show that educated women and husbands, women residing in the urban area, and the wealthiest households are more likely to utilize C-section delivery in the Sub-Saharan region. Additionally, the higher percentage of births attended by skilled health staff of a country can significantly reduce the C-section delivery for variables educational attainment (respondent & husband), place of residence, and wealth index, exhibited by meta-regression. Although maternal, neonatal, and infant mortality and morbidity can be influenced by unnecessary C-sections, it can save women and their new-born lives in case of life-threatening pregnancy and childbirth-related complications. Therefore, our results suggest that strategies are needed to provide C-sections through proper facilities for rural, uneducated, impoverished Sub-Saharan African women to minimize maternal and infant mortality so that they can get access to the lifesaving procedure when necessary. Furthermore, the significant role of skilled health staff in the delivery period must be illustrated among pregnant women by health-related programs in each Sub-Saharan African country.

## Supplementary Information

Supplementary Information 1.

Supplementary Information 2.

Supplementary Information 3.

## Data Availability

Data are available at https://dhsprogram.com/.
