# Supplementary material for: Caesarean delivery and its association with educational attainment, wealth index, and place of residence in Sub-Saharan Africa: a meta-analysis
Source: Sci Rep. 2022 Apr 1;12:5554. doi: 10.1038/s41598-022-09567-1 (PMC8975863; doi:10.1038/s41598-022-09567-1)
Supplement: Supplementary file 1 — Supplementary Information 1. [file 41598_2022_9567_MOESM1_ESM.pdf]

Caesarean delivery and its association with educational attainment, wealth index, and place of residence in Sub-Saharan Africa: a meta-analysis

Md. Akhtarul Islam*[a], Nusrat Jahan Sathi[a], Md. Tanvir Hossain[b], Abdul Jabbar[c], Andre M.N. Renzaho[d#], Sheikh Mohammed Shariful Islam[e#]

[a] Statistics Discipline, Science, Engineering and Technology School, Khulna University, Khulna 9208, Bangladesh.
[b] Sociology Discipline, Social Science School, Khulna University, Khulna 9208, Bangladesh.
[c] Department of Clinical Medicine, Faculty of Veterinary Science, University of Veterinary and Animal Sciences, Lahore Punjab Pakistan
[d] School of Social Sciences, Western Sydney University, Penrith 2751 NSW Australia
[e] Institute for Physical Activity and Nutrition, Deakin University, Melbourne, Victoria 3125, Australia

#Equal contribution

*Corresponding author

Md. Akhtarul Islam
Statistics Discipline
Science, Engineering and Technology School,
Khulna University, Khulna 9208, Bangladesh
akhtarulstat@ku.ac.bd
https://orcid.org/0000-0003-2396-2168
+8801722736875

Word count: 3145

The appendix (1-4) shows the overall effect estimate changes after removing one study. The overall effect size was calculated, omitting one country at a time. The leave-one-out analysis reported evidence that Ethiopia, Nigeria, and Congo Democratic Republic had greatest influence on pooled estimated of caesarian section. From Appendix 1, omitting Ethiopia reduces the overall effect size 4.11% (95% CI: 2.70-3.49). Omitting each country does not influence the overall effect size, presented in Appendix 2. Appendix 3 exhibited that the pooled effect size is smallest (2.44) while omitting Nigeria. In Appendix 4, removing Nigeria decrease the overall effect size and removing Congo Democratic Republic increase the pooled effect size.

Appendix 1 Results of Sensitivity Analysis for Place of Residence

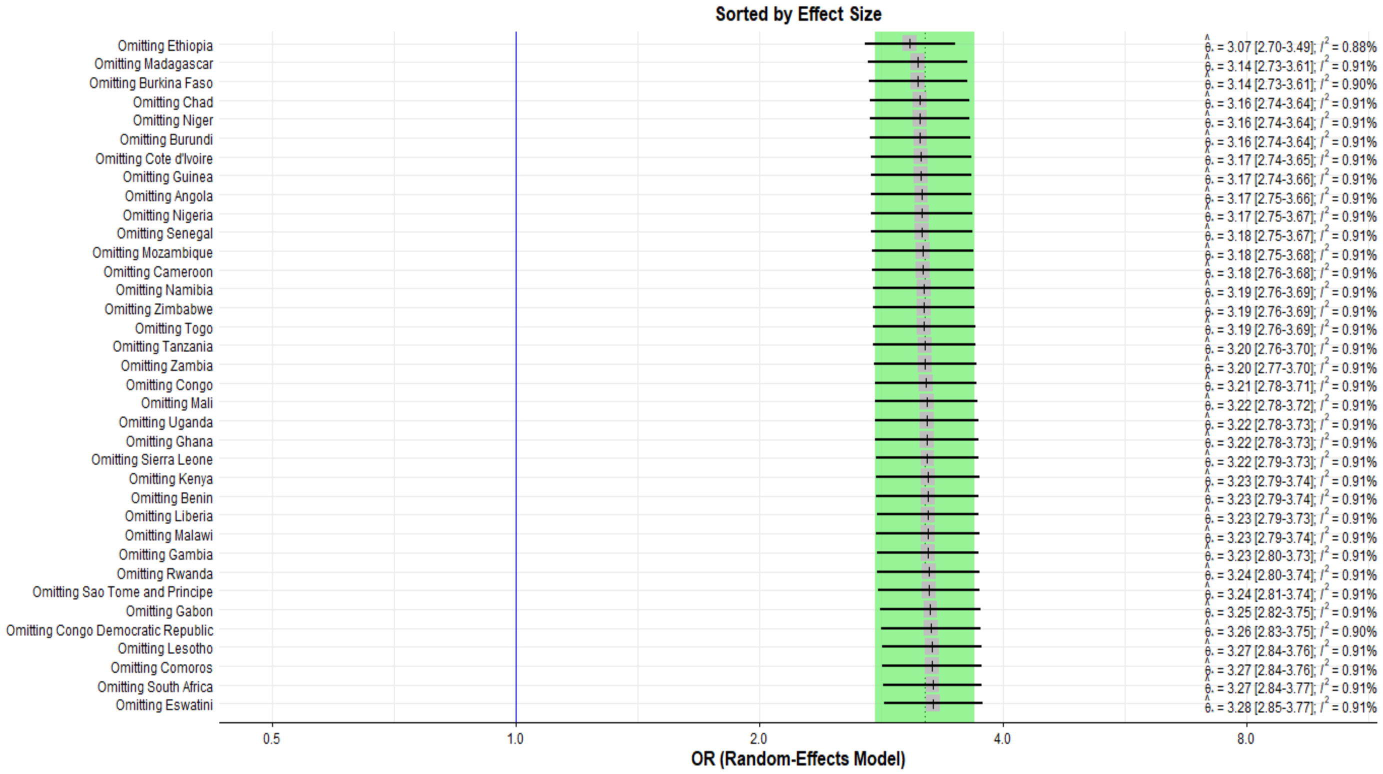

Influence of each country on the overall pooled estimate from leave-one-country-out sensitivity analysis

Appendix 2 Results of Sensitivity Analysis for Wealth Index

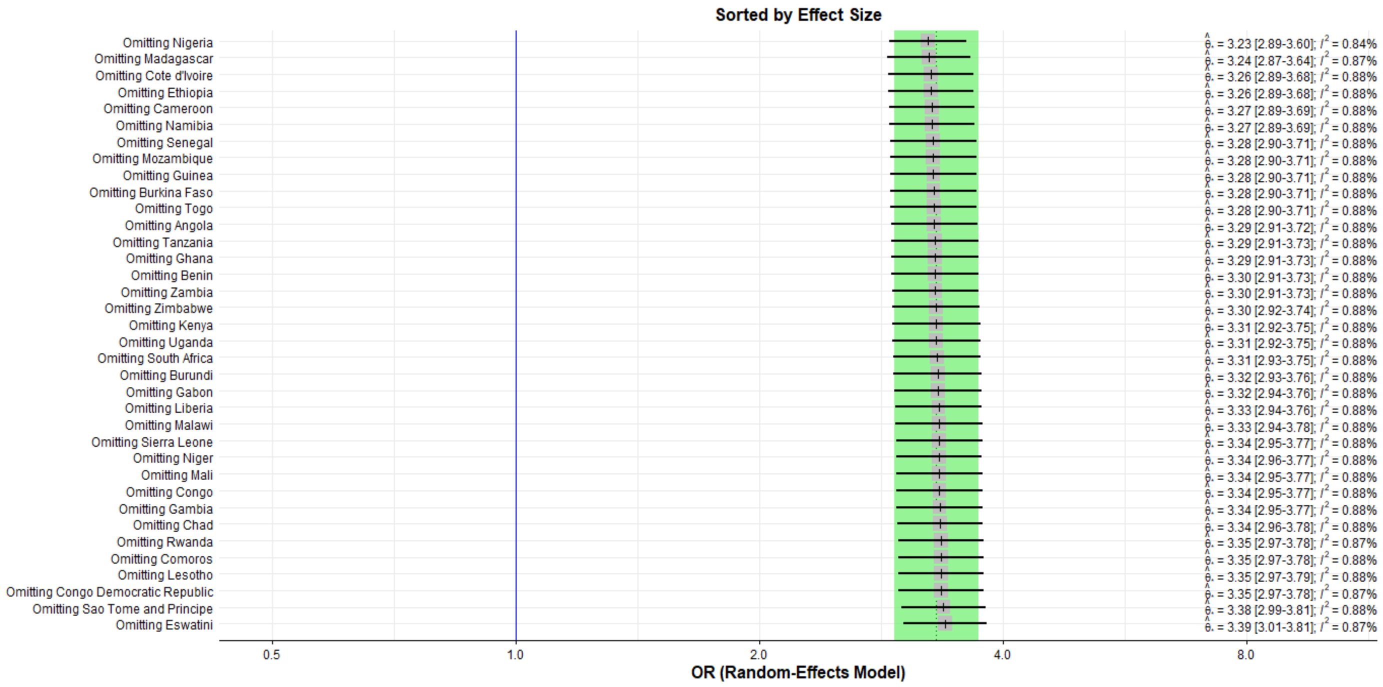

Influence of each country on the overall pooled estimate from leave-one-country-out sensitivity analysis

Appendix 3 Results of Sensitivity Analysis for Women Education

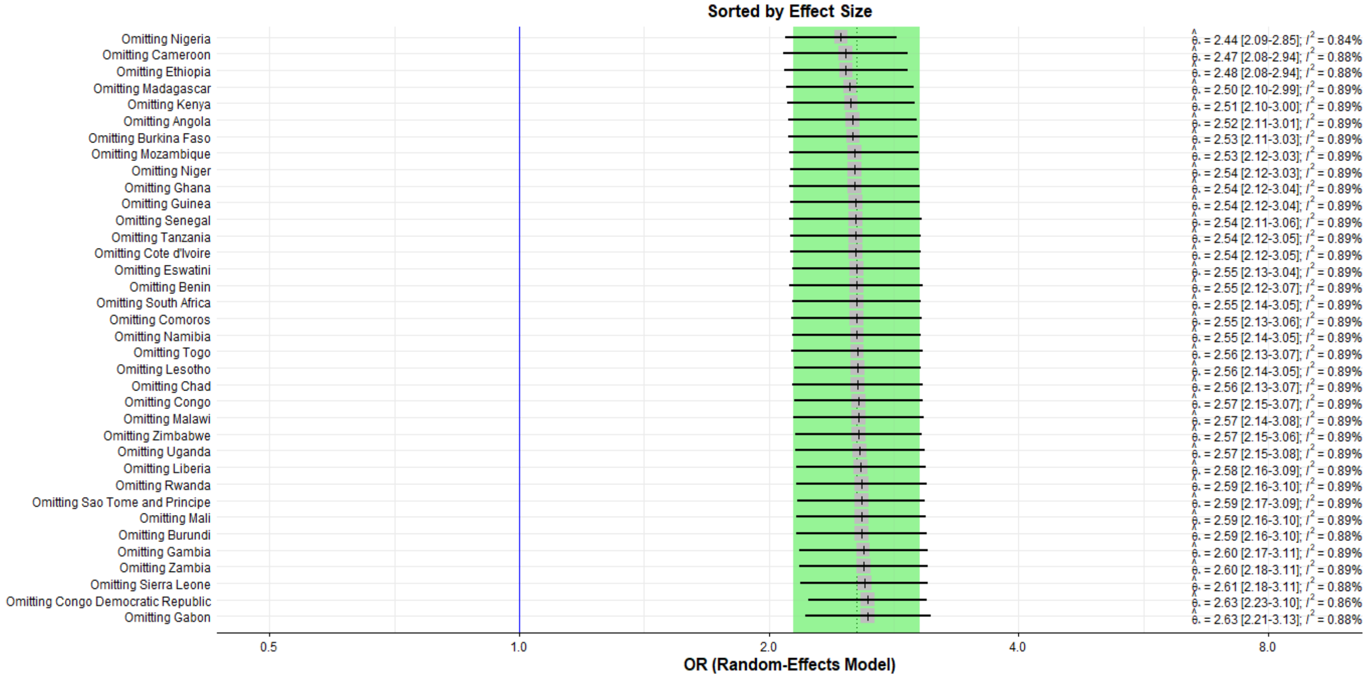

Influence of each country on the overall pooled estimate from leave-one-country-out sensitivity analysis

Appendix 4 Results of Sensitivity Analysis for Husband Education

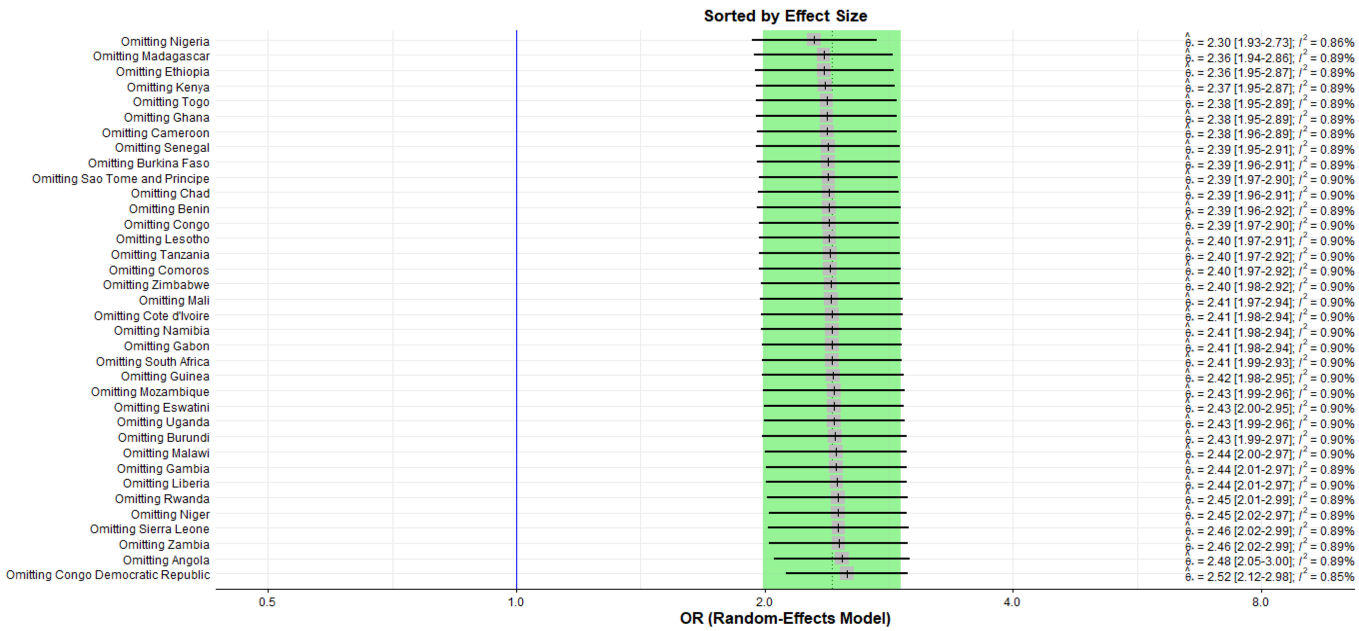

Influence of each country on the overall pooled estimate from leave-one-country-out sensitivity analysis
